# The Effects of Leptin on Breastfeeding Behaviour

**DOI:** 10.3390/ijerph121012340

**Published:** 2015-09-30

**Authors:** Anna M. Cannon, Foteini Kakulas, Anna R. Hepworth, Ching Tat Lai, Peter E. Hartmann, Donna T. Geddes

**Affiliations:** School of Chemistry and Biochemistry, Faculty of Science, The University of Western Australia, Perth, Western Australia 6009, Australia; E-Mails: anna.cannon@research.uwa.edu.au (A.M.C.); foteini.kakulas@uwa.edu.au (F.K.); anna.hepworth@uwa.edu.au (A.R.H.); ching-tat.lai@uwa.edu.au (C.T.L.); peter.hartmann@uwa.edu.au (P.E.H.)

**Keywords:** leptin, human milk, breast milk, breastfeeding, appetite regulation, lactation

## Abstract

Breastfed infants have a reduced risk of becoming overweight and/or obese later in life. This protective effect has been partly attributed to leptin present in breastmilk. This study investigated 24-h variations of skim milk leptin and its relationship with breastmilk macronutrients and infant breastfeeding patterns. Exclusive breastfeeding mothers of term singletons (*n* = 19; age 10 ± 5 weeks) collected pre- and post-feed breastmilk samples for every breastfeed over a 24-h period and test-weighed their infants to determine milk intake at every breastfeed over a 24-h period. Samples (*n* = 454) were analysed for leptin, protein, lactose and fat content. Skim milk leptin concentration did not change with feeding (*p* = 0.184). However, larger feed volumes (>105 g) were associated with a decrease in post-feed leptin levels (*p* = 0.009). There was no relationship between the change in leptin levels and change in protein (*p* = 0.313) or lactose levels (*p* = 0.587) between pre- and post-feed milk, but there was a trend for a positive association with changes in milk fat content (*p* = 0.056). Leptin concentration significantly increased at night (*p* < 0.001) indicating a possible 24-h pattern. Leptin dose (ng) was not associated with the time between feeds (*p* = 0.232). Further research should include analysis of whole breastmilk and other breastmilk fractions to extend these findings.

## 1. Introduction

Breastfeeding is critical for the optimal growth, protection and development of the term infant. Longer breastfeeding periods have been associated with reduced risk of the infant becoming overweight and/or obese later in life and developing non-communicable diseases (NCDs) [1,2,3,4,5,6,7,8,9]. Infants allowed to breastfeed on demand develop better control of appetite and feeding behaviours when introduced to solid foods than formula-fed infants [10,11]. These effects have been mainly attributed to the composition of human milk, particularly the presence of appetite control hormones, although the mode of feeding has also been implicated [12,13,14].

Breastmilk leptin has been the most widely studied appetite hormone, and a strong association between breastmilk leptin and a reduced risk of developing obesity later in life has been shown [1,2,11,15]. Leptin has therefore been considered to be integral to appetite regulation and energy balance in breastfed infants [2]. Breastfeeding on demand facilitates self-regulation of the feed volume by the infant [16,17,18] and cessation of the feed generally indicates fullness and satiety [17,19]. Given the negative association between breastmilk leptin and infant growth [1,20], it is likely that breastmilk leptin may influence the frequency and/or milk volume taken by the infant. This has never been investigated despite the reduced risk of obesity and other NCDs breastfeeding is known to confer. Our study aimed to determine any changes in skim milk leptin levels during a breastfeed; whether these changes, if any, were associated with the levels of breastmilk macronutrients; and to explore the relationship of skim milk leptin with 24-h breastfeeding patterns in term infants.

## 2. Experimental Section

### 2.1. Ethics Statement

All procedures involving human subjects were approved by the Human Research Ethics Committee of The University of Western Australia. Mothers provided informed written consent to participate in the study (RA/4/1/4253).

### 2.2. Participants

Breastfeeding mother-infant dyads were recruited through the Australian Breastfeeding Association and Community Health Centres between 2006 and 2013 for ongoing studies. Selection criteria for this study included: exclusive breastmilk feeding; infant age less than 24 weeks; singleton; and born full term (37–40 weeks [21]) and sufficient sample volume to do the biochemical analysis. Exclusion criteria included: maternal breast surgeries and piercings, as these are known to affect milk production [22]; and mothers with 24-h milk productions outside the normal range of 440–1220 mL [17]. Demographic information of the participants was also collected. Maternal Body Mass Index (BMI) was determined using standard calculation for metric units (weight divided by height squared) (Table 1). 

**Table 1:** Characteristics of the study cohort (*n* = 19 breastfeeding dyads).

Mother and Infant Characteristics	Mean ± SD	Range
Mother		
age (years)	32 ± 3	27–37
BMI *	25 ± 4	18–33
parity	NA	1–3
Infant		
age (weeks)	10 ± 5	3–21
birth weight (g)	3515 ± 416	2930–4325
current weight (g)	5912 ± 1296	4062–8990

Abbreviations: BMI—body mass index; * BMI was calculated for *n* = 18.

### 2.3. Sample Collection

Mothers test-weighed their infants using electronic scales (BabyWeigh Scale, Medela Inc, McHenry, IL, USA resolution 2 g, accuracy; ±0.034%) before and after each breastfeed during a 24-h period plus one breastfeeding. They also hand-expressed small samples of breastmilk (<5 mL) from each breast into polypropylene plastic vials (Disposable Products, Adelaide, Australia) before and after each breastfeed. Mothers labelled the samples and placed them immediately in the home freezer (−20 °C). When sample collection was completed, vials were transported to the laboratory on ice and stored at −20 °C until biochemical analyses [17]. Total 24-h milk production was determined as previously described [23]. 

### 2.4. Biochemical Analyses

Prior to analysis, samples were thawed at room temperature (RT) and aliquoted into 1.5 mL tubes (Sarstedt, Numbrecht, Germany). Skim milk was obtained by centrifugation at RT in a Beckman Microfuge 11 (Aberdon Enterprise Inc., IL, USA) at 7537 × g for 10 min. The fat layer was removed by clipping it off together with the top of the tube. Fat concentration of whole milk was determined within 3–5 days of the sample arrival at the laboratory by the creamatocrit method [24] using the Creamatocrit Plus™ device (Medela Inc., McHenry, IL, USA). Fat content was calculated from the cream content of the milk samples based on the equation: 5.917 × cream percentage + 3.56, and expressed in g/L [25]. All skim milk samples were analysed for protein, lactose and leptin concentrations.

Protein concentration was measured using the Bradford Protein Assay adapted from Mitoulas *et al.* [26], with a detection limit of 0.049 g/L and an inter-assay CV of 15.8% (*n* = 13). Lactose concentration was determined using the enzymatic-spectrophotometric method of Kuhn and Lowenstein [27] adapted from Mitoulas, Kent, Cox, Owens, Sherriff and Hartmann [26], with a detection limit of 2.37 g/L and an inter-assay CV of 5.7% (*n* = 13).

Leptin in skim breastmilk was determined by an enzyme linked immunosorbent assay (ELISA) using the Human Leptin DuoSet kit (R&D Systems, Minneapolis, MN, USA), which was optimised to measure leptin in skim breastmilk. Skim milk samples and quality control samples were sonicated by an ultrasonic processor VCX130 (Sonics & Material, Newton, CT, USA). For this, the milk sample was placed on ice and sonicated using maximum power (100%), 3 bursts at 5 s each, with 20 s cooling intervals. Sonicated samples were then diluted 1:10 with 1% w/v BSA in PBS (pH 7.4). Standards (recombinant human leptin Part 840281) were diluted in 1% w/v BSA in PBS in the concentration range of 0.0–0.9 ng/mL. Briefly, 96-well EIA/RIA plates (Corning, Union City, CA, USA) were coated with 100 μL/well of capture antibody (mouse anti-human leptin Part 840279; working concentration of 4 μg/mL in PBS, pH 7.4) and incubated overnight at RT. The next day the plate was washed in wash buffer (0.05% Tween 20 in PBS, pH 7.4) using a plate washer (model 1575, Bio-Rad Laboratories, Hercules, CA, USA), and 300 μL/well of blocking buffer (1% w/v BSA in PBS, pH 7.4) were applied. The plate was incubated at RT for 1 h and then washed in wash buffer. Subsequently, 100 μL of sample, standard or QC were assayed in duplicate. The plate was incubated at RT for 2 h and washed. Detection antibody was added at 100 μL/well (biotinylated mouse anti-human leptin Part 840280; working concentration of 25 ng/mL in 1% w/v BSA in PBS, pH 7.4) and the plate was incubated at RT for 2 h. The plate was then washed in wash buffer, and 100 μL/well of Streptavidin–HRP (1:200 in 1% w/v BSA in PBS, pH 7.4) were applied followed by a 20-min incubation at RT. The plate was then washed again and 100 μL/well of colour reagent (1:1 mixture of Colour Reagent A, H_2_O_2_, and Colour Reagent B, Tetramethylbenzidine) were applied. The plate was incubated at RT for 15 min in the dark and 50 μL/well of 3 M H_2_SO_4_ were added. Absorbance readings were taken at 450 nm, 540 nm and 570 nm (2 readings at 5-min intervals) using a plate spectrophotometer Power Wave XS Microplate reader (BioTek, Winooski, VT, USA). The latter two wavelengths were used to correct any optical imperfections of the plate. Recovery of a known amount of leptin added to the skim milk samples was 98.4% ± 6.8% (*n* = 13). The detection limit of the assay was 0.017 ng/mL and the inter-assay CV was 9.9%.

### 2.5. Leptin Dose and Intake

Leptin dose was defined as the amount of leptin ingested with a given volume of breastmilk during a feed from one breast. If the infant breastfed from more than one breast with less than 30-min interval between breasts, that was considered to be one feeding session [17]. To calculate leptin dose from one breast, the mean of the pre- and post-feed leptin concentration was multiplied by the corresponding feed volume. In case of feeding from more than one breast, leptin dose of separate breasts was summed. Twenty-four hour leptin intake was calculated as the sum of all feed leptin doses that occurred in the 24-h period.

### 2.6. Statistical Analysis

Statistical analysis was performed in R 2.9.01 [28] for Mac OSX. Additional packages nlme [29] and lattice [30] were used for linear mixed effect models and graphical presentation of the data, respectively. Package MuMIn [31] was used to calculate conditional *R*^2^ which describes the proportion of variance explained fixed and random effects used in the linear mixed effect models. Descriptive statistics are reported as mean ± SD unless otherwise stated. *p* ≤ 0.05 was considered statistically significant.

Due to low sample volume, composition data were missing for the following milk samples: leptin, lactose, protein (*n* = 7); fat (*n* = 11) across 9 participants. Feed volumes (*n* = 2), expression volumes (*n* = 2), and maternal weight and height values (*n* = 1 participant) were not recorded. Where data were missing, available case analysis was used.

Responses were modelled using linear mixed effects models (grouping by participant, or breast within participant) or linear regression as appropriate, as determined using ANOVA to compare models with the same fixed effects.

Changes in leptin concentration were evaluated using the above-mentioned statistical approach with further fixed effect of feed volumes. To further explore the relationship between pre- and post-feed leptin levels, change and feed volumes, feed volumes were grouped into first quartile (0–41 g; *n* = 67), second quartile (42–65 g; *n* = 57), third quartile (66–105 g; *n* = 62) and fourth quartile (>105 g; *n* = 56), and linear regression analysis of feed volume quartiles was modelled (Section 3.1.2).

To assess variability of leptin concentration across the participants, a linear mixed effects model was used with fixed effect of leptin concentration and a random effect of different baseline change per individual. Differences in pre- and post-feed concentrations of leptin, fat, protein and lactose were compared against zero. Any associations between difference in leptin concentration and macronutrient content (fat, protein and lactose) were tested using linear regression (Section 3.1.3). 

The relationship between leptin dose and feeding behaviours was modelled with meal volume and time interval to the next feed as the responses of interest, and leptin dose as the fixed effect. Random effects of different baselines per individual, and per breast within individual were considered. To account for possible effects of meal duration and volume on the time to the next feed, these were included as covariates in the model (Section 3.1.4).

To further explore changes in leptin, protein and lactose concentrations over a 24-h period, we considered the proposed rhythm of changes in blood leptin [32] and the fact that mothers breastfed on demand in this study. Based on the above, the 24-h period was divided into four time periods of 6 h as follows: morning (4:01 AM to 10:00 AM), day (10:01 AM to 4:00 PM), evening (4:01 PM to 10:00 PM), and night (10:01 PM to 4:00 AM) [17,33]. Linear regression with fixed effect of time periods was used to evaluate changes in leptin, protein and lactose concentrations between the time periods (morning/day/evening/night). Tukey’s multiple comparisons were made for leptin, protein and lactose concentrations and the four time periods (Section 3.1.5).

Associations between leptin (24-h intake or concentration and demographic characteristics (24-h milk intake, maternal BMI, infant age, birth weight, current weight, and infant gender) were examined using a linear mixed effects model with the random effect of participant baseline and breast baseline. Fixed effects of leptin and demographic characteristics were tested in each model in 2- and 3-way interactions. Fixed effects of macronutrients, maternal BMI, pre-/post-feed leptin concentration and total milk intake were tested (Section 3.1.6).

## 3. Results and Discussion

### 3.1. Results

#### 3.1.1. Participants

The characteristics of the infants are given in Table 1. All infants (*n* = 19) were healthy and growing appropriately for their age according to World Health Organization’s growth charts for breastfed infants [34]. 

#### 3.1.2. Variability of Skim Milk Leptin

**Figure 1:**
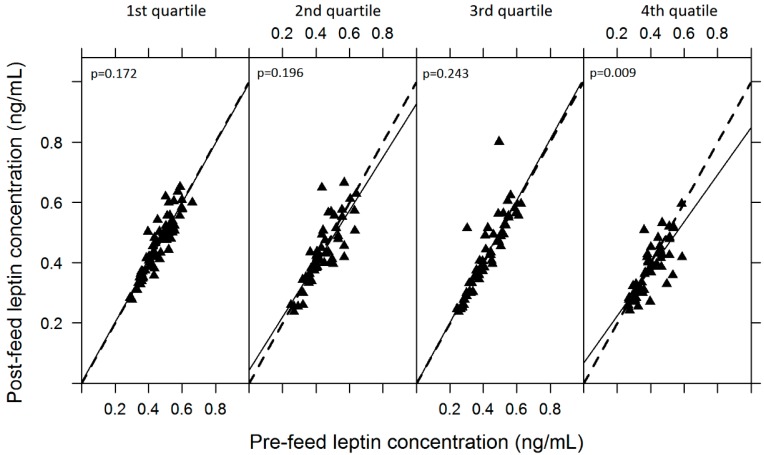
Relationship between the pre- and post-feed skim milk leptin levels and feed volume, where feed volumes are divided into the first quartile (0–41 g; *n* = 67), the second quartile (42–65 g; *n* = 57), the third quartile (66–105 g; *n* = 62) and the fourth quartile 105 g; *n* = 56). Regression and equivalence lines are shown in solid black and dashed lines, respectively.

The average leptin concentration in skim breastmilk was 0.43 ± 0.10 ng/mL (range: 0.26–0.57 ng/mL) (Table 2) with no overall difference between the left and right breasts (*p* = 0.999) and different leptin levels were observed between individuals (*p* < 0.001). There was no significant difference between pre- and post-feed leptin levels (*p* = 0.184). However, there was a significant association between changes in pre- and post-feed leptin levels and feed volume (*p* = 0.014). Further analysis of feed volumes involved dividing them into quartiles and revealed that in 16 out of 19 participants feed volumes of more than 105 g were recorded. These large feeds were associated with a significantly greater decrease in post-feed leptin levels (fourth quartile; *p* = 0.009), with an average decrease of 0.02 ng per 1 mL of skim milk (Figure 1). 

**Table 2:** Breastmilk concentration of protein, lactose, fat and leptin in 19 breastfeeding dyads over a 24-h period, measured in pre- and post-feed breastmilk samples (mean ± SD). The *p*-values refer to the difference tested between pre- and post-feed values for these breastmilk components.

Breastmilk Constituents	Pre-Feed Value	Post-Feed Value	N Samples	*p*-Value
Protein (g/L)	11.18 ± 1.67	11.33 ± 1.94	483	0.14
Lactose (g/L)	67.04 ± 6.03	66.04 ± 5.89	483	0.001
Fat (g/L)	31.37 ± 11.95	56.95 ± 20.19	479	<0.0001
Leptin (ng/mL)	0.43 ± 0.10	0.42 ± 0.11	479	0.184

#### 3.1.3. Relationship of Skim Milk Leptin with Macronutrients

The overall differences between pre- and post-feed protein, lactose and fat are shown in Table 2, with lactose (*p* = 0.001) being significantly lower and fat (*p* < 0.001) levels being significantly higher in the post-feed samples by 1.1 g and 25 g on average, respectively. There was a positive trend between the pre- and post-feed change in fat and the magnitude of change between pre- and post-feed skim milk leptin levels (*p* = 0.056), where the greater the difference in pre- and post-feed leptin the greater the difference in milk fat content. There was no relationship between the pre- and post-feed change in leptin and the change in protein (*p* = 0.313) or lactose levels (*p* = 0.587), respectively.

#### 3.1.4. Skim Milk Leptin and Breastfeeding Patterns

The average dose of skim milk leptin in a feed and total leptin intake over a 24-h period were 45.94 ± 21.25 ng (*n* = 19, range: 9.62–104.84) and 403.36 ± 80.08 ng (*n* = 19, range: 239.64–501.40), respectively (Table 3). There was a positive association between leptin dose and feed volume (*p* < 0.001) between individuals. Analysis revealed different individual patterns for participants with lower and higher leptin levels (*p* < 0.001). There was no overall significant association between leptin dose and the time between feeds (*p* = 0.232) or the duration of the feed (*p* = 0.08), and feed volume (*p* = 0.177) did not affect the association. 

**Table 3:** Breastmilk production and infant feeding characteristics over a 24-h period for 19 breastfeeding dyads (Mean ± SD).

Breastmilk and Feeding Characteristics	Mean ± SD	Range
Total production of both breasts (mL)	822 ± 166	496–1232
Left breast production (mL)	373 ± 140	89–666
Right breast production (mL)	434 ± 119	259–676
Number of feeds *	9 ± 2	7–13
Volume of feeds (mL) *	108 ± 45	18–220
Feed duration (min) *	24 ± 13	4–105
Feed interval (min) **	172 ± 102	23–590
Skim milk leptin dose per feed (ng) *	45.94 ± 21.25	9.62–104.84
Skim milk leptin intake (ng) ***	403.36 ± 80.08	239.64–501.40

* If the infant breastfed from more than one breast, with less than 30-min interval between breasts, this was considered to be one feeding session [17]. ** From the beginning of one feed to the beginning of the following feed; when the infant feeds from one breast and the following feed is more than 30 min later [17]. *** Total skim milk leptin dose over a 24-h period.

#### 3.1.5. Twenty-Four Hour Pattern of Skim Milk Leptin and Macronutrients

**Figure 2:**
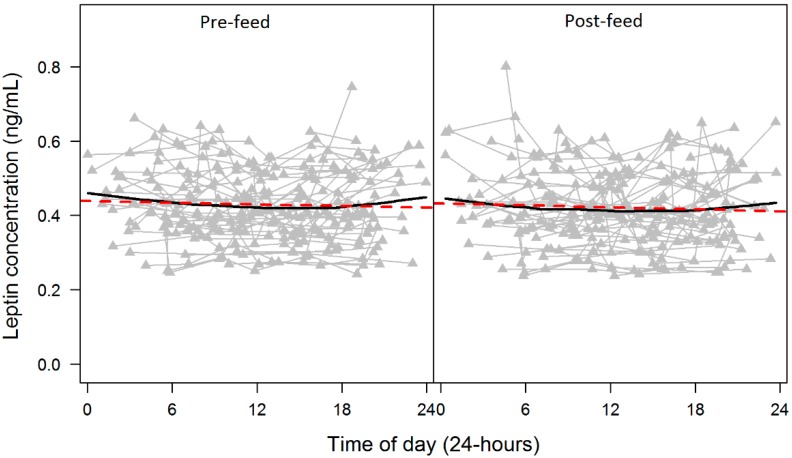
Changes in skim milk leptin concentration over a 24-h period for pre- and post-feed skim milk breastmilk samples. Standard regression line (**red line**) and LOESS local regression smoother (**black line**) indicate the general pattern of change across individual time points (**grey lines**).

Skim milk leptin showed subtle variations across a 24-h period in both pre- and post-feed samples. Leptin levels gradually decreased between 00:00 and 06:00, with a plateau between ~06:00 and 17:00, and then increased until 24:00 (Figure 2). Analysis of leptin concentration at 4 different time periods showed that leptin levels were significantly higher during 10 PM to 4 AM (22:01–04:00) compared to 10 AM to 4 PM (10:01–16:00; *p* = 0.004) and to 4 PM to 10 PM (16:01–22:00; *p* = 0.002).

Protein and lactose concentrations showed large variation between pre- and post-feed milk samples across a 24-h period, with no consistent patterns, although lactose was significantly lower between 4 PM and 10 PM (16:01–22:00) compared to 10 AM and 4 PM (10:01–16:00; *p* < 0.001). The milk fat content showed a continuous increase in pre-feed samples until ~20:00 and slowly plateaued. Post-feed samples showed a sharp increase between 00:00 and 12:00, with the evident peak ~13:00, and a slow decline until 24:00. Feed volumes decreased between 00:00 and ~12:00, and did not change until 24:00.

#### 3.1.6. Skim Milk Leptin and Demographics

Associations between leptin and demographic characteristics are presented in Table 4. For each additional unit of maternal BMI, there was a decrease in average fat concentration (by 0.9 g/L) and an increase in leptin (by 0.013 ng/mL) and protein concentrations (by 0.1 g/L).

### 3.2. Discussion

Leptin is well known to influence short- and long-term appetite control in adults; however its function in infants is poorly understood. This study investigated factors that may be associated with changes in skim milk leptin levels over a 24-h period in fully breastfeeding mother-infant dyads. Initiation rates are high in Australian and West Australian women (90.4%) with approximately 60% exclusively breastfed at 1 month, further declining to approximately 40% at 4 months and 15% at 6 months [35,36]. Therefore, we are often able to recruit fully breastfeeding mothers to research studies and given the relatively high breastfeeding rate compared to other countries such as US [37], our sample is considered representative of the Australian population in term of the duration of exclusive breastfeeding. Further, the benefits of breastfeeding are dose dependent [1,2,3,7,12] and therefore fully breastfed infants represent both the evolutionary norm and infants that will most benefit from breastfeeding.

**Table 4:** Demographic characteristics of the breastfeeding dyads (*n* = 19) and associations with skim milk leptin concentration (ng/mL) and total skim leptin intake (ng) over a 24-h period. Data is presented with *p*-values and conditional *R*^2^ for univariate and multivariate models.

Demographic Characteristics	Leptin Concentration (ng/mL) *p*-Value	Conditional *R*^2^ for Univariate ^1^ and Multivariate ^2^ Models	Total Leptin Intake (ng/24-h) *p*-Value	Conditional *R*^2^ for Univariate ^1^ and Multivariate ^2^ Models
Maternal				
BMI	0.008 ^1^	0.77 ^1^	0.394 ^1^	0.05 ^1^
breast left or right	0.619 ^1^	0.77 ^1^	N/A	N/A
pre-/post-feed sample	0.317 ^2^	0.77 ^2^	N/A	N/A
total milk intake	0.595 ^2^	0.78 ^2^	N/A	N/A
Infant age (weeks)	0.638 ^1^	0.78 ^1^	0.21 ^1^	0.09 ^1^
Infant current weight (g)	0.769 ^1^	0.78 ^1^	0.067 ^1^	0.19 ^1^
Infant gender	0.220 ^1^	0.78 ^1^	0.616 ^1^	0.02 ^1^
Total milk intake (mL/24 h)	0.394 ^1^	0.78 ^1^	0.002 ^1^	0.45 ^1^

*p*-values and conditional *R*^2^ for univariate ^**1**^ and multivariate ^**2**^ models.

Skim milk leptin concentration was comparable with some of the studies that also analysed skim breastmilk but used radioimmunoassay (RIA) (0.20 to 0.50 ng/mL) [38,39,40]. In contrast, leptin concentrations in studies which analysed skim milk by ELISA were somewhat lower than ours (0.16 to 0.48 ng/mL) [1,18,41,42]. A handful of studies measured leptin concentration in whole milk using RIA reporting values 3–170 times higher than in our study (1.34 to 73.22 ng/mL) [43,44,45,46,47]. Such variability in results is partly due to the interference between milk fat and RIA [39,46].

Changes in pre- and post-feed skim milk leptin levels were insignificant overall in this study, which agrees with previous studies that evaluated breastmilk leptin changes [18,38,42]. While those studies examined changes for a single feed, here we profiled changes in leptin levels across a 24-h period, reinforcing the reliability of absence of change across a feed. Although there was no overall change in leptin levels, a significant decrease in post-feed samples (up to 18%) was observed when feed volumes were greater than 105 g. The average breastfeed volume and 24-h milk production have been shown to not change within a breastfeeding infant between 1 and 6 months of lactation [17,26,48]. The only behaviour shown to change is an increase in efficiency of milk removal, in that milk transfer increases and feed duration decreases [48]. Further, there is a wide range in volumes taken by an infant over a 24-h period. It is estimated that 24.3% of infants never take feed larger than 105 g. Whereas 18.6% of infants have 1 feed more than 105 g and 27.1%, 14.3%, 11.4%, 4.3% have 2,3,4,5 feeds greater than 105 g (derived from data presented in [17]). Our findings therefore suggest that when exploring relationships between leptin and other variables, pre- and post-feed sample collection as well as measuring infant feed volume should be considered. 

Changes in skim milk leptin levels were not associated with changes in either protein or lactose levels. However, there was a trend with the change in milk fat levels, where a greater difference in fat was associated with a greater difference in leptin. A similar relationship was also observed where larger feeds were associated with a larger decrease in leptin. Milk fat content is known to be higher in post-feed samples, increasing upon emptying of the breast, and larger feeds tend to have a higher fat content [49,50]. However, milk fat content of the actual feed has not been related to feed volume or the time interval between feeds [17]. This suggests the possibility of an intermediary relationship between leptin, fat and feed volume, which could be further elucidated by analysis of whole breastmilk. Indeed, leptin has been previously reported to be higher in whole milk [40,43,45,47], which could be due to its postulated association with the milk fat globule [43] or with the cell fraction of breastmilk [12], and merits further investigation. 

Breastfeeding patterns among infants are highly variable in terms of frequency of feeding, feed volume, and breast preference (left or right) [17,26]. Mothers recruited in this study were representative of the Australian mothers in terms of duration of the exclusive breastfeeding [35,36]. Concentration of protein, lactose and fat (Table 2) and the breastfeeding and milk production characteristics of the women in this study (Table 3) agree with previously reported values [17,26,33], with the exception of skim milk leptin dose and 24-h leptin intake, which have not been previously reported. The skim milk leptin dose correlated with feed volume, such that larger feed volumes delivered more leptin to the infant. Interestingly, mothers with lower breastmilk leptin levels tended to have smaller changes in the pre- to post-feed leptin levels as opposed to mothers with higher leptin levels, which tended to have greater changes across the feed. Different leptin levels observed between mothers may be partly explained by differences in the maternal BMI, which positively correlated with leptin (Table 3), meaning that the higher the BMI in a woman/mother the higher her leptin levels are [1,39,43]. Statistical analysis in this cross-sectional study showed that there was no relationship between leptin levels and infant age, which was not entirely unexpected (Table 4). Longitudinal studies have shown differences in leptin concentrations between colostrum (2–3 days postpartum), transitional milk (3–5 days postpartum) and milk during established lactation (3.5–6 weeks postpartum) [47]. Further, a cross-sectional study has shown a decrease in milk leptin concentration up to 180 days. However, there were no significant differences between 3 groups of women between 25 and 180 days of lactation [51]. Our infants were between the ages of 3 to 21 weeks, therefore lie within the range of ages that previous studies have shown no difference in leptin levels. Since fully breastfed infants consume approximately the same daily volume of milk during this period and exhibit similar feeding frequencies, it is not surprising that milk leptin levels did not change. It has also been speculated that early feeding practices program infant appetite and leptin likely has a role in this event [12]. 

We did not find any association between the leptin dose of a feed and the time to the next feed. However, individual positive and negative correlations were observed, suggesting that for some infants a feed of a greater leptin dose was followed by a longer interval before the next breastfeed, while other infants initiated a feed in a relatively short period (Figure 2). It also agrees with previous research which found that intervals between feeds differ among infants, with a wide range of intervals of 1 to 8 h following a relatively small (~35 mL) or large (~175 mL) feeds [17]. Interestingly, 3 week old infant had 6 (out of 9) feeds larger than 105 g while 6 week old infant with 25% lower 24-h milk intake had only 1 (out of 8) feed larger than 105 g; oldest infant (21 weeks of age) had 5 (out of 8) feeds larger than 105 g and 24-h milk intake similar to the 3 week old infant. This implies the possibility of individual relationships, which is likely influenced by a number of factors such as maternal BMI (Table 4) or changes in fat and cell contents [50]. In this context, it must be noted that whole milk leptin has not been investigated over a 24-h period and that satiety is affected by a plethora of other appetite hormones present in breastmilk, which were not measured here. 

In contrast to other components of milk such as protein or lactose [26,33], skim milk leptin displayed a subtle 24-h pattern, with a significant rise during the period 10 PM to 4 AM, which agrees with the previously reported nocturnal rise in human blood leptin [32,52,53,54] and visceral adipose tissue leptin in rats [55,56]. Despite being an anorexigenic hormone released into the blood in response to food intake with short- and long-term effects on feeding behaviour [57], leptin in human blood does not appear to be stimulated by changes in glucose and insulin levels or meal ingestion [32]. It is therefore unlikely that the maternal meal schedule would influence leptin levels in breastmilk However, clarification is required by simultaneous recording of maternal food intake over a 24-h period and collection of milk and maternal blood samples at appropriate time points. Further, it is possible that breastmilk leptin might be influenced by the maternal brain in particular the arcuate nucleus of the hypothalamus [58], which highly expresses the leptin receptor and contains neurons that respond directly to leptin [59]. Given the relationship between milk leptin and maternal blood leptin, it is possible that both plasma leptin and skim milk leptin levels are regulated in the same manner [1,42,43]. Moreover, leptin variations could also be associated with breast fullness, as we have found a positive trend between change in leptin and change in fat across 24-h [17,50,60]. Therefore, a relationship between variation of fat and leptin cannot be excluded until leptin in whole milk, and its individual fractions are examined.

The 24-h pattern of leptin in human milk should be further explored in terms of other potentially influencing factors, such as signaling/regulation pathways and their relationship with non-communicable diseases (NCDs). Research has demonstrated the importance of leptin for the normal development of brain tissue in neonates by promoting neural growth and the development of the hypothalamic circuitry and acting on the arcuate nucleus of the hypothalamus [61,62]. These findings suggest that limited access to leptin during the postnatal period could contribute to childhood obesity [11,61]. It is worth noting that artificial infant formula lacks leptin [63]. This opens another avenue of research to elucidate the effects of breastmilk leptin on infant development, breastfeeding behaviour, obesity risk, and the short- and long-term dietary patterns of infants.

### 3.3. Limitations

Nineteen women were included in this study where intensive sampling was performed over 24-h period. While numbers may be regarded as small, this is the first time changes in leptin have been measured extensively over the day. Whilst the samples were stored for an extended period of time, all biochemical measurements were comparable with the literature. Investigation into the effects of storage times and temperatures on milk appetite hormones such as leptin however is required. This study is limited to the full breastfeeding period of the first 6 months of life, however it does represent the breastfeeding “norm”. Replications of these studies for whole milk are required to confirm the results of this study.

## 4. Conclusions

Infants exclusively breastfed on demand demonstrate a wide range of self-regulated feeding patterns, with long-term benefits in preventing obesity, however factors influencing appetite control in these infants are not well understood. Leptin levels in skim breastmilk did not change upon milk removal unless the volume was larger than 105 g. Leptin in skim milk does not appear to have an influence on breastfeeding patterns, however fluctuations suggest a potential 24-h pattern that merits further investigation. These findings require confirmation in whole milk over extended periods of time.

## References

[1] Miralles O., Sanchez J., Palou A., Pico C. (2006). A physiological role of breast milk leptin in body weight control in developing infants. Obesity.

[2] Palou A., Pico C. (2009). Leptin intake during lactation prevents obesity and affects food intake and food preferences in later life. Appetite.

[3] Geddes D.T., Prescott S.L. (2013). Developmental origins of health and disease: The role of human milk in preventing disease in the 21st century. J. Hum. Lact..

[4] Von Kries R., Koletzko B., Sauerwald T., von Mutius E., Barnert D., Grunert V., von Voss H. (1999). Breast feeding and obesity: Cross sectional study. BMJ.

[5] Gillman M.W., Rifas-Shiman S.L., Camargo C.A., Berkey C.S., Frazier A.L., Rockett H.R., Field A.E., Colditz G.A. (2001). Risk of overweight among adolescents who were breastfed as infants. JAMA.

[6] Bergmann K.E., Bergmann R.L., von Kries R., Bohm O., Richter R., Dudenhausen J.W., Wahn U. (2003). Early determinants of childhood overweight and adiposity in a birth cohort study: Role of breast-feeding. Int. J. Obes. Relat. Metab. Disord..

[7] McCrory C., Layte R. (2012). Breastfeeding and risk of overweight and obesity at nine-years of age. Soc. Sci. Med..

[8] Owen C.G., Martin R.M., Whincup P.H., Smith G.D., Cook D.G. (2005). Effect of infant feeding on the risk of obesity across the life course: A quantitative review of published evidence. Pediatrics.

[9] Armstrong J., Reilly J.J., Child Health Information Team (2002). Breastfeeding and lowering the risk of childhood obesity. Lancet.

[10] Savino F., Liguori S.A., Fissore M.F., Oggero R. (2009). Breast milk hormones and their protective effect on obesity. Int. J. Pediatr. Endocinol..

[11] Stocker C.J., Cawthorne M.A. (2008). The influence of leptin on early life programming of obesity. Trends Biotechnol..

[12] Hassiotou F., Geddes D.T. (2014). Programming of appetite control during breastfeeding as a preventative strategy against the obesity epidemic. J. Hum. Lact..

[13] Li R., Magadia J., Fein S.B., Grummer-Strawn L.M. (2012). Risk of bottle-feeding for rapid weight gain during the first year of life. Arch. Pediatr. Adolesc. Med..

[14] Bartok C.J. (2011). Babies fed breastmilk by breast *versus* by bottle: A pilot study evaluationg early growth patterns. Breastfeed. Med..

[15] Savino F., Costamagna M., Prino A., Oggero R., Silvestro L. (2002). Leptin levels in breast-fed and formula-fed infants. Acta Paediatr..

[16] Dewey K.G., Heinig M.J., Nommsen L.A., Lonnerdal B. (1991). Maternal *versus* infant factors related to breast milk intake and residual milk volume: The darling study. Pediatrics.

[17] Kent J.C., Mitoulas L.R., Cregan M.D., Ramsay D.T., Doherty D.A., Hartmann P.E. (2006). Volume and frequency of breastfeedings and fat content of breastmilk throughout the day. Pediatrics.

[18] Karatas Z., Durmus Aydogdu S., Dinleyici E.C., Colak O., Dogruel N. (2011). Breastmilk ghrelin, leptin, and fat levels changing foremilk to hindmilk: Is that important for self-control of feeding?. Eur. J. Pediatr..

[19] Bartok C.J., Ventura A.K. (2009). Mechanisms underlying the association between breastfeeding and obesity. Int. J. Pediatr. Obes..

[20] Schuster S., Hechler C., Gebauer C., Kiess W., Kratzsch J. (2011). Leptin in maternal serum and breast milk: Association with infants’ body weight gain in a longitudinal study over 6 months of lactation. Pediatr. Res..

[21] Hale T.W., Hartmann P.E. (2007). Hale and Hartmann’s Textbook of Human Lactation.

[22] Kent J.C., Prime D.K., Garbin C.P. (2011). Principles for maintaining or increasing breast milk production. J. Obstet. Gynecol. Neonatal. Nurs..

[23] Arthur P.G., Hartmann P.E., Smith M. (1987). Measurement of the milk intake of breast-fed infants. J. Pediatr. Gastroenterol. Nutr..

[24] Fleet I.R., Linzell J.L. (1964). A rapid method of estimating fat in very small quantities of milk. J. Physiol..

[25] Meier P.P., Engstrom J.L., Zuleger J.L., Motykowski J.E., Vasan U., Meier W.A., Hartmann P.E., Williams T.M. (2006). Accuracy of a user-friendly centrifuge for measuring creamatocrits on mothers’ milk in the clinical setting. Breastfeed. Med..

[26] Mitoulas L.R., Kent J.C., Cox D.B., Owens R.A., Sherriff J.L., Hartmann P.E. (2002). Variation in fat, lactose and protein in human milk over 24 h and throughout the first year of lactation. Br. J. Nutr..

[27] Kuhn N.J., Lowenstein J.M. (1967). Lactogenesis in the rat. Changes in metabolic parameters at parturition. Biochem. J..

[28] R Core Team (2009). R: A Language and Environment for Statistical Computing.

[29] Pinheiro J., Bates D., DebRoy S., Sarkar D., R Core Team (2009). Nlme: Linear and Nonlinear Mix Effects Models.

[30] Sarkar D. (2008). Lattice: Multivariate Data Visualization with R.

[31] Burnham K.P., Anderson D.R. (2002). Model Selection and Multimodel Inference: A Practical Information-Theoretic Approach.

[32] Sinha M.K., Ohannesian J.P., Heiman M.L., Kriauciunas A., Stephens T.W., Magosin S., Marco C., Caro J.F. (1996). Nocturnal rise of leptin in lean, obese, and non-insulin-dependent diabetes mellitus subjects. J. Clin. Investig..

[33] Khan S., Hepworth A.R., Prime D.K., Lai C.T., Trengove N.J., Hartmann P.E. (2013). Variation in fat, lactose, and protein composition in breast milk over 24 h: Associations with infant feeding patterns. J. Hum. Lact..

[34] WHO The Who Child Growth Standards. http://www.who.int/childgrowth/standards/en/.

[35] Australian Institute of Health and Walfare (2011). 2010 Australian National Infant Feeding Survey: Indicator Results.

[36] Forde K.A., Miller L.J. (2010). 2007–2009 North metropolitan perth breastfeeding cohort study: How long are mothers breastfeeding?. Breastfeed. Rev..

[37] Centers for Disease Control and Prevention (2014). Breastfeeding Report Card.

[38] Schueler J., Alexander B., Hart A.M., Austin K., Larson-Meyer D.E. (2013). Presence and dynamics of leptin, glp-1, and pyy in human breast milk at early postpartum. Obesity.

[39] Eilers E., Ziska T., Harder T., Plagemann A., Obladen M., Loui A. (2011). Leptin determination in colostrum and early human milk from mothers of preterm and term infants. Early Hum. Dev..

[40] Uysal F.K., Onal E.E., Aral Y.Z., Adam B., Dilmen U., Ardicolu Y. (2002). Breast milk leptin: Its relationship to maternal and infant adiposity. Clin. Nutr..

[41] Bronsky J., Mitrova K., Karpisek M., Mazoch J., Durilova M., Fisarkova B., Stechova K., Prusa R., Nevoral J. (2011). Adiponectin, AFABP, and leptin in human breast milk during 12 months of lactation. J. Pediatr. Gastroenterol. Nutr..

[42] Weyermann M., Beermann C., Brenner H., Rothenbacher D. (2006). Adiponectin and leptin in maternal serum, cord blood, and breast milk. Clin. Chem..

[43] Houseknecht K.L., McGuire M.K., Potrocarrero C.P., McGuire M.A., Beerman K. (1997). Leptin is present in human milk and is related to maternal plasma leptin concentration and adiposity. Biochem. Biophys. Res. Commun..

[44] Smith-Kirwin S.M., O’Connor D.M., De Johnston J., Lancey E.D., Hassink S.G., Funanage V.L. (1998). Leptin expression in human mammary epithelial cells and breast milk. J. Clin. Endocrinol. Metab..

[45] Ucar B., Kirel B., Bor O., Kilic F.S., Dogruel N., Aydogdu S.D., Tekin N. (2000). Breast milk leptin concentrations in initial and terminal milk samples: Relationships to maternal and infant plasma leptin concentrations, adiposity, serum glucose, insulin, lipid and lipoprotein levels. J. Pediatr. Endocrinol. Metab..

[46] Lonnerdal B., Havel P.J. (2000). Serum leptin concentrations in infants: Effects of diet, sex, and adiposity. Am. J. Clin. Nutr..

[47] Bielicki J., Huch R., von Mandach U. (2004). Time-course of leptin levels in term and preterm human milk. Eur. J. Endocrinol..

[48] Sakalidis V.S., Kent J.C., Garbin C.P., Hepworth A.R., Hartmann P.E., Geddes D.T. (2013). Longitudinal changes in suck-swallow-breathe, oxygen saturation, and heart rate patterns in term breastfeeding infants. J. Hum. Lact..

[49] Daly S.E., di Rosso A., Owens R.A., Hartmann P.E. (1993). Degree of breast emptying explains changes in the fat content, but not fatty acid composition, of human milk. Exp. Physiol..

[50] Hassiotou F., Hepworth A.R., Williams T.M., Twigger A.J., Perrella S., Lai C.T., Filgueira L., Geddes D.T., Hartmann P.E. (2013). Breastmilk cell and fat contents respond similarly to removal of breastmilk by the infant. PLoS ONE.

[51] Ilcol Y.O., Hizli Z.B., Ozkan T. (2006). Leptin concentration in breast milk and its relationship to duration of lactation and hormonal status. Int. Breastfeed. J..

[52] Langendonk J.G., Pijl H., Toornvliet A.C., Burggraaf J., Frolich M., Schoemaker R.C., Doornbos J., Cohen A.F., Meinders A.E. (1998). Circadian rhythm of plasma leptin levels in upper and lower body obese women: Influence of body fat distribution and weight loss. J. Clin. Endocrinol. Metab..

[53] Matkovic V., Ilich J.Z., Badenhop N.E., Skugor M., Clairmont A., Klisovic D., Landoll J.D. (1997). Gain in body fat is inversely related to the nocturnal rise in serum leptin level in young females. J. Clin. Endocrinol. Metab..

[54] Laughlin G.A., Yen S.S. (1997). Hypoleptinemia in women athletes: Absence of a diurnal rhythm with amenorrhea. J. Clin. Endocrinol. Metab..

[55] Ando H., Yanagihara H., Hayashi Y., Obi Y., Tsuruoka S., Takamura T., Kaneko S., Fujimura A. (2005). Rhythmic messenger ribonucleic acid expression of clock genes and adipocytokines in mouse visceral adipose tissue. Endocrinology.

[56] Bodosi B., Gardi J., Hajdu I., Szentirmai E., Obal F., Krueger J.M. (2004). Rhythms of ghrelin, leptin, and sleep in rats: Effects of the normal diurnal cycle, restricted feeding, and sleep deprivation. Am. J. Physiol. Regul. Integr. Comp. Physiol..

[57] Pico C., Oliver P., Sanchez J., Palou A. (2003). Gastric leptin: A putative role in the short-term regulation of food intake. Br. J. Nutr..

[58] Huang W., Ramsey K.M., Marcheva B., Bass J. (2011). Circadian rhythms, sleep, and metabolism. J. Clin. Investig..

[59] Bouret S.G., Simerly R.B. (2007). Development of leptin-sensitive circuits. J. Neuroendocrinol..

[60] Khan S., Prime D.K., Hepworth A.R., Lai C.T., Trengove N.J., Hartmann P.E. (2013). Investigation of short-term variations in term breast milk composition during repeated breast expression sessions. J. Hum. Lact..

[61] Bouret S.G., Simerly R.B. (2004). Minireview: Leptin and development of hypothalamic feeding circuits. Endocrinology.

[62] Vickers M.H., Sloboda D.M. (2012). Leptin as mediator of the effects of developmental programming. Best Pract. Res. Clin. Endocrinol. Metab..

[63] Savino F., Benetti S., Liguori S.A., Sorrenti M., Cordero D.I.M.L. (2013). Advances on human milk hormones and protection against obesity. Cell Mol. Biol..

